# Clozapine-induced, dilated cardiomyopathy: a case report

**DOI:** 10.1186/s13104-017-2679-5

**Published:** 2017-07-27

**Authors:** Sarah Longhi, Stephan Heres

**Affiliations:** Klinik und Poliklinik für Psychiatrie und Psychotherapie, Klinikum rechts der Isar der TU München, Ismaningerstr.22, 81675 Munich, Germany

**Keywords:** Clozapine, Cardiomyopathy, Side-effect, Psychosis, Schizophrenia, Case report

## Abstract

**Background:**

Clozapine is known to be a highly effective antipsychotic agent and additionally provides a significant reduction in suicide-risk and aggression. Clozapine-induced cardiomyopathy is a very rare but nonetheless dangerous side-effect, with an incidence of 0.02–0.1%, depending on the literature, and a mortality rate of up to 17.9%.

**Case presentation:**

We report on the case of a 25 year-old patient, who was admitted for the evaluation of a potential electroconvulsive therapy due to persistent auditory hallucinations under clozapine. Shortly after admission he was found to be suffering from dilated cardiomyopathy, likely caused by his antipsychotic treatment.

**Conclusion:**

Clozapine-induced cardiomyopathy should be taken into account when monitoring patients treated with this antipsychotic and regular electrocardiograms should be performed in order to detect possible alterations as soon as possible.

## Background

Clozapine is known to be a highly effective antipsychotic agent and additionally provides a significant reduction in suicide-risk and aggression [6, 9–11]. Since some of its side-effects, e.g. agranulocytosis, which occurs in nearly 1% of all treated patients, can be life-threatening clozapine is not considered a first-line antipsychotic and should only be administered after two failed treatment attempts with other antipsychotics [2]. Psychiatrists tend to focus on the well-known side effects like agranulocytosis and the development of metabolic syndrome when monitoring a clozapine-treated patient, yet the cardiac risk should not be underestimated as we try to demonstrate in our case report.

For example, Haas and colleagues published a review of 116 cases of clozapine-associated myocarditis identified in an Australian database [5].

Furthermore, it is worth mentioning, that agranulocytosis first came into focus as a clozapine-related side-effect in a Finnish case report in 1975, thus stressing the importance of singular case reports in the process of understanding the possible risks of pharmacological treatment [7].

Clozapine-induced cardiomyopathy is a very rare but nonetheless dangerous side-effect, with an incidence of 0.02–0.1%, depending on the literature, and a mortality rate of up to 17.9% [4, 8]. According to the manufacturers last published report of 2002, which was also the most recent made available to us, 178 cases have been reported worldwide, of which 32 patients died. Thus a re-challenge with clozapine of a patient who previously has experienced a clozapine-induced myocarditis or cardiomyopathy is strongly discouraged, as discussed in the review of Nielsen et al. in 2013 [12].

In this article we report on the case of a 25 year-old patient, who was admitted for the evaluation of a potential electroconvulsive therapy (ECT) due to persistent auditory hallucinations under clozapine. Shortly after admission he was found to be suffering from dilated cardiomyopathy, likely caused by his antipsychotic treatment.

## Case presentation

The patient presented in January 2015 with auditory hallucinations, i.e. voices telling him, that he had raped a girl a few years back. The patient was first diagnosed with paranoid schizophrenia in 2013 and had been treated with various antipsychotics, including aripiprazole, haloperidol, amisulpride, flupenthixol and risperidone without any significant effect on his symptoms. On admission his medication consisted of 900 mg clozapine, 800 mg amisulpride and 10 mg flupenthixol. He was living and working in a sheltered facility, was under regular medical care by a psychiatrist as well as a general practitioner and was well integrated in his family.

**Fig. 1:**
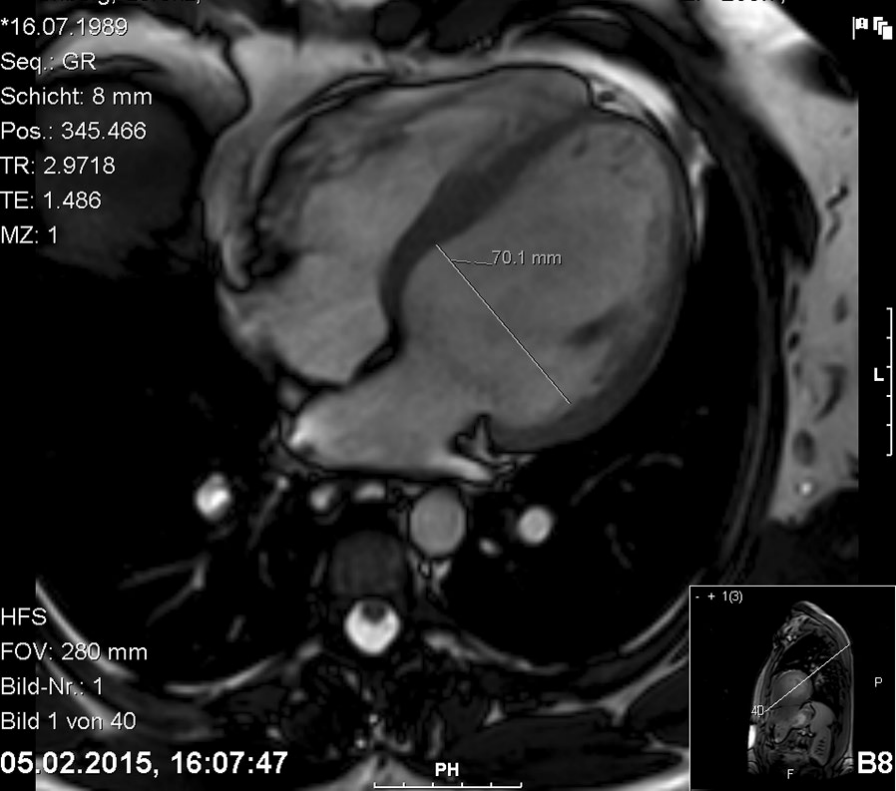
Cardiac MRI, left ventricle with a diameter of 70.1 mm

The patient complained of shortness of breath, palpitations and general fatigue, symptoms that his parents and the treating psychiatrist had mainly interpreted as negative symptoms such as anhedonia, flat affect and social withdrawal. The patient had been suffering from these symptoms for about 6 months prior to admission. In the electrocardiogram (ECG) obtained on admission we noted sinus tachycardia, bigeminy and a prolonged QTc-interval at 484 ms. The echocardiogram showed signs of dilated cardiomyopathy with severe left ventricular systolic dysfunction (ejection fraction down at 33%). These findings were confirmed in a cardiac MRI showing a dilated left ventricle (diameter 70.1 mm, see Fig. 1) and no signs of ischemia/post-ischemia or inflammation. The cardiologists recommended treatment for heart-failure with an ACE-inhibitor, a β-blocker and a diuretic agent as well as the immediate discontinuation of clozapine, the most likely cause of the cardiac impairment according to the cardiologists. Furthermore they considered the patient not suitable for ECT and scheduled a reevaluation of the patient’s cardiac status for 4 weeks later.

The literature-search for the best alternative antipsychotic medication with a better cardiac-risk profile yet yielding high antipsychotic efficacy resulted in olanzapine, which had been recommended in a recent review as well as in a similar case report [1, 3]. Together with the patient and after consulting the manufacturer we decided not to abruptly discontinue the clozapine-treatment but to taper it off in six steps each 150 mg per day while starting with olanzapine and escalating the dose up to 30 mg under daily ECG-control. Olanzapine was titrated over 6 days with daily dose increments of 5 mg. Flupenthixol was discontinued as well in order to minimize QTc-prolongation. The patient fortunately did not experience a significant worsening of psychotic symptoms; in fact he even reported a slight improvement of the auditory hallucinations regarding frequency and intensity under treatment with 30 mg olanzapine per day.

Alas with more or less unchanged ECGs (bigeminy, couplets, triplets, QTc-interval between 440 and 490 ms) and clinical manifestations, i.e. shortness of breath, fatigue, palpitations, the cardiologists did not note a significant improvement of cardiac function after 4 weeks. The echocardiogram still showed severe left ventricular systolic dysfunction (ejection fraction 35%) and the patient could not be cleared for ECT. They recommended an additional treatment with spironolactone and considered the prophylactic implantation of an implantable cardioverter-defibrillator (ICD) if the following 2 months would not result in a significant improvement.

We discharged the patient on an antipsychotic medication of 30 mg olanzapine and 800 mg amisulpride.

When the patient returned for the cardiologic follow-up visit in July 2015, the ECG had slightly improved, but the echocardiogram remained unchanged; another visit in 3 months was recommended. The psychopathology on the other hand had improved further; the patient seemed remarkably more stable and active than on the first admission.

In the latest cardiologic follow-up (October 2015) the ECG showed no signs of cardiac impairment, with a normal sinus rhythm at a rate of 67 bpm and a QTc-interval of 440 ms. Moreover, the echocardiogram was greatly improved; the ejection fraction had risen to 54% and the patient himself confirmed that the initial clinical symptoms of impending heart failure were no longer present.

## Discussion and conclusions

Though being extremely rare and occurring roughly 10 times less often than the commonly feared agranulocytosis (incidence 0.1% versus 1%), clozapine- induced cardiomyopathy should be taken into account when monitoring patients treated with this antipsychotic and regular ECGs should be performed.

It should also be noted, that one hypothesis concerning the underlying pathomechanism causing dilated cardiomyopathy under clozapine-therapy is an untreated, acute myocarditis, which mainly occurs in the first 14–21 days after the beginning of treatment [5, 8]. One possible solution could be to implement a more thorough monitoring protocol for the first 4 weeks of treatment in order to detect changes in cardiac function in a timely manner. Ronaldson et al. for example recommended a protocol which suggested an echocardiography at baseline as well as measurement of troponin I/T and CRP at baseline and day 7, 14, 21 and 28 of treatment. In contrast, the clozapine cardiac monitoring guidelines circulated by Novartis Australia and Hospira Australia, only suggested an ECG and the measurement of troponin I/T and serum creatinine at baseline and further controls of these parameters on day 7 and 14 [13].

If, as we described in our case, the patient already presents with the typical clinical manifestations of heart failure such as shortness of breath, palpitations, ventricular arrhythmia or fatigue, further diagnostic measures should be taken in a timely manner to rule out severe cardiac impairment, especially since recovery is possible.

Those symptoms should not be considered as part of negative symptoms or a post-schizophrenic depressive episode.

## Abbreviations

ECT: electroconvulsive therapy
ECG: electrocardiogram
ICD: implantable cardioverter-defibrillator
CRP: C-reactive protein
